# Multisensory Integration-Attention Trade-Off in Cochlear-Implanted Deaf Individuals

**DOI:** 10.3389/fnins.2021.683804

**Published:** 2021-07-29

**Authors:** Luuk P. H. van de Rijt, A. John van Opstal, Marc M. van Wanrooij

**Affiliations:** ^1^Department of Otorhinolaryngology, Donders Institute for Brain, Cognition and Behaviour, Radboudumc, Nijmegen, Netherlands; ^2^Department of Biophysics, Donders Institute for Brain, Cognition and Behaviour, Radboud University, Nijmegen, Netherlands

**Keywords:** multisensory integration, focused attention, divided attention, cochlear implant, audiovisual, speech perception

## Abstract

The cochlear implant (CI) allows profoundly deaf individuals to partially recover hearing. Still, due to the coarse acoustic information provided by the implant, CI users have considerable difficulties in recognizing speech, especially in noisy environments. CI users therefore rely heavily on visual cues to augment speech recognition, more so than normal-hearing individuals. However, it is unknown how attention to one (focused) or both (divided) modalities plays a role in multisensory speech recognition. Here we show that unisensory speech listening and reading were negatively impacted in divided-attention tasks for CI users—but not for normal-hearing individuals. Our psychophysical experiments revealed that, as expected, listening thresholds were consistently better for the normal-hearing, while lipreading thresholds were largely similar for the two groups. Moreover, audiovisual speech recognition for normal-hearing individuals could be described well by probabilistic summation of auditory and visual speech recognition, while CI users were better integrators than expected from statistical facilitation alone. Our results suggest that this benefit in integration comes at a cost. Unisensory speech recognition is degraded for CI users when attention needs to be divided across modalities. We conjecture that CI users exhibit an integration-attention trade-off. They focus solely on a single modality during focused-attention tasks, but need to divide their limited attentional resources in situations with uncertainty about the upcoming stimulus modality. We argue that in order to determine the benefit of a CI for speech recognition, situational factors need to be discounted by presenting speech in realistic or complex audiovisual environments.

## Introduction

Speech recognition is a challenging task. First, the speech signal itself might be hard to recognize due to poor pronunciation, variable spectral and temporal cues across talkers (such as fundamental frequency, formant frequencies, and voice onset time), semantic ambiguities and highly variable and rapid articulation rates (>200 words/min; Miller, 1982). Second, in common everyday environments, even highly salient speech signals are frequently embedded in acoustic background noise and are masked by other talkers. During face-to-face conversation, non-acoustic cues from seeing a talker’s mouth can improve speech recognition in those situations, through the integration of visual and auditory information (Sumby and Pollack, 1954; Summerfield, 1992; Bernstein et al., 2004; Helfer and Freyman, 2005; Peelle and Sommers, 2015).

Multisensory integration is beneficial for normal-hearing and normally sighted individuals, whenever multisensory stimuli are in spatial-temporal congruence. The effects of audiovisual integration are clearly evident from goal-directed behavior and include behavioral benefits, such as shorter reaction times (Corneil et al., 2002; Bremen et al., 2017; Colonius and Diederich, 2017), increased localization accuracy and precision (Corneil et al., 2002; Alais and Burr, 2004), and reduced ambiguity (McDonald et al., 2000). These behavioral effects are typically reflected by enhanced neuronal activity (Stein and Meredith, 1993; van de Rijt et al., 2016; Colonius and Diederich, 2017). This also applies to more complex auditory stimuli; supplemental visual input enhances speech perception, and audiovisual speech recognition embedded in noise is considerably better than for auditory speech alone (MacLeod and Summerfield, 1987; Sommers et al., 2005; Ross et al., 2006; Bosen et al., 2018). Even when stimuli are incongruent, a visual influence on auditory perception can be observed in illusory phenomena, such as the McGurk effect (McGurk and MacDonald, 1976) and the ventriloquist effect (Jack and Thurlow, 1973; Alais and Burr, 2004). The necessity to integrate non-acoustic information to improve performance becomes especially clear for individuals with hearing impairments, such as profoundly deaf individuals using a cochlear implant (CI). The CI typically recovers hearing to an extent that allows the CI user to understand speech in quiet situations, yet performs poorly under more challenging listening conditions (e.g., noisy surroundings). In these cases, the CI user should rely more on the information obtained from lip reading. Evidence suggests that CI users are indeed better able to integrate visual information with the degraded acoustic information than normal-hearing individuals (Schorr et al., 2005; Rouger et al., 2007).

Due to all the observed benefits of multisensory integration, one may forget that it requires paying attention to multiple sensory modalities at the same time. Attention is a neural mechanism by which the brain is able to effectively select a relevant signal from a multitude of competing sources (e.g., finding someone with a red coat in a busy street). When attention is fully focused on a particular sensory modality, say auditory, performance in auditory selection tasks will markedly increase, but visual stimuli will likely be missed, because attention has limited capacity. The opposite occurs when attention is focused on vision. In natural environments, however, the most relevant sensory modality of a potential target may not be known in advance, and therefore focusing attention on a single sensory modality may not be an optimal strategy to maximize perceptual performance. Instead, in such cases, attention should be divided across the relevant modalities. In case of speech perception, these modalities are auditory (listening) and visual (lipreading) signals. Dividing attention across modalities will allow the brain to integrate the multimodal signals when they originate from the same source, and filter out the distracting background from unrelated sources.

However, because of its limited capacity, dividing attention in an uncertain sensory environment may lead to decreased performance for stimuli that happen to be unisensory, as each modality will receive less attentional amplification than during a fully focused attention task. Here we compared word-recognition performance during focused and divided attention tasks users and normal-hearing individuals, by presenting unisensory and/or bi-sensory spoken sentences in different sensory-noise regimes. Because CI users have more difficulty to process the degraded auditory input, more effort (i.e., more attention) will be required to understand auditory speech. Therefore, we reasoned that in a divided-attention task, the reduced attention to audition (and vision) may lead to poorer unisensory performance scores in CI users. In principle, the same reasoning may hold for normal-hearing participants. So far, it remains unclear from the literature whether CI users can successfully divide their attention across modalities, and whether divided attention affects their speech-recognition abilities.

## Materials and Methods

### Participants

Fourteen native Dutch-speaking, normal-hearing participants (mean age: 22.3 years ±1.8, 10 female) and seven native Dutch-speaking, post-lingually deaf unilaterally implanted CI users (mean age 64.1 years ±5.3, 3 female) were recruited to participate in this study. All CI users had at least 1 year of experience with their CI, with a mean of 3.6 years ±1.8. Five CI users were implanted on the left. The cause of deafness was progressive sensorineural hearing loss for all but three CI users (Ménière’s disease, sudden deafness and hereditary hearing loss). Additional contralateral hearing aids were turned off during the experiment. The unaided pure tone average (range 1–4 kHz) of the non-implanted ear ranged between 70 and >120 dB Hearing Loss. However, no CI users had any speech intelligibility for words in quiet with their non-implanted ear at levels <90 dB Sound Pressure Level (SPL). All normal-hearing participants were screened for normal hearing (within 20 dB HL range 0.5–8 kHz). All participants reported normal or corrected-to-normal vision. All participants gave written informed consent before taking part in the study. The experiments were carried out in accordance with the relevant institutional and national regulations and with the World Medical Association Helsinki Declaration as revised in October 2013. The experiments were approved by the Ethics Committee of Arnhem-Nijmegen (project number NL24364.091.08, October 18, 2011).

### Stimuli

The audiovisual material was based on the Dutch version of the speech-in-noise matrix test developed by Houben et al. (2014). In general, a matrix test uses sentences of identical grammatical structure in which all available words are taken from a closed set of alternatives. The sentences are syntactically fixed (subject, verb, numeral, adjective, object), but semantically unpredictable.

**FIGURE 1:**
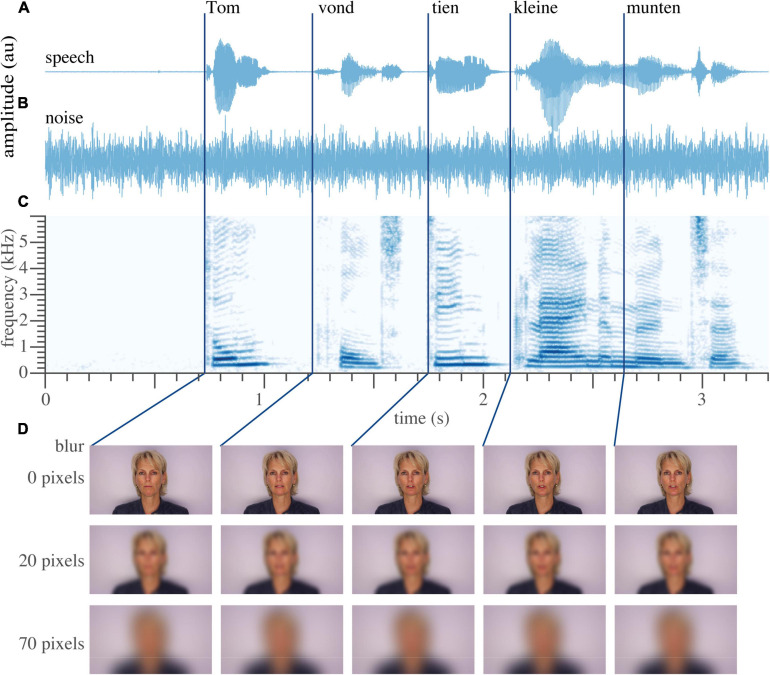
Example sentence. **(A)** Temporal waveform of the auditory speech signal “Tom vond tien kleine munten” (translation: Tom found 10 little coins.) **(B)** Waveform of the auditory noise. **(C)** Spectrogram of the recorded sentence. **(D)** Five video frames around the onset of the word, untouched (top), moderately blurred (middle, 20 pixels), and extensively blurred (bottom, 70 pixels, used as a unisensory auditory condition in the divided-attention task). Dark blue lines denote the approximate onset of each individual word. Written informed consent for the publication of this image was obtained from the individual shown.

The audiovisual material (Figure 1) including the masking speech-shaped noise is reported previously van de Rijt et al. (2019). Briefly, the stimulus material consisted of digital video recordings of a female speaker reading aloud the sentences in Dutch. Auditory speech (Figures 1A,C) was presented with varying levels of acoustic background noise (Figure 1B). Visual speech consisted of the video fragments of the female speaker (Figure 1D). Saliency of the visual speech was altered through blurring, by filtering every image of the video with a 2-D Gaussian smoothing kernel at several pixel standard deviations.

### Set-Up

The experiments were performed in an experimental room, in which the walls and ceiling were covered with black acoustic foam that eliminated echoes for sound frequencies >500 Hz (Agterberg et al., 2011). Stimulus presentation was controlled by a Dell PC (Dell Inc., Round Rock, TX, United States) running Matlab version 2014b (The Mathworks, Natick, MA, United States). Participants were seated in a chair 1 m in front of a PC screen (Dell LCD monitor, model: E2314Hf). Sounds were played through an external PC sound card (Babyface, RME, Germany) and presented through one speaker (Tannoy, model Reveal 502) placed above the PC screen, 1 m in front of the participant (30° above the interaural plane). Speaker level was measured with an ISO-TECH Sound Level Meter, type SLM 1352P at the position of the participant’s head, using the masking noise.

### Paradigm

All participants were tested on a closed set of 6 Matrix lists of 20 sentences of 5 words each. Participants were instructed to select words from the Matrix list which they recognized.

### Familiarization

To familiarize participants with the Matrix test procedure and to obtain an initial estimate for the auditory threshold, 40 unique auditory-only sentences were presented. The signal-to-noise ratio varied adaptively in accordance with the Brand and Kollmeier procedure (Brand and Kollmeier, 2002) and the auditory 50% speech recognition threshold was calculated as the average signal-to-noise ratio of the last nine sentences. This threshold was used to individualize the signal-to-noise ratios in focused-attention experiment. For normal-hearing participants, the noise level was fixed, while for the CI users the speech level was fixed, both at 60 dB(A). This was also true for both experiments.

### Focused-Attention Task

In this experiment participants listened to auditory-only sentences in one block and viewed visual-only sentences in another block. The participants were asked to accurately indicate the words heard after each sentence (10-alternative, open-ended choice; 10 alternatives were available for each word, but participants were allowed to not pick any alternative). Each trial was self-paced. Participants either heard 60 (for the first three participants) or 40 unique sentences in each block.

In the auditory-only block, the auditory speech was presented in acoustic background noise with uninformative visual input (i.e., a black screen for six normal-hearing participants; or a heavily blurred video (70 pixel blur) for eight normal-hearing participants and all CI users; no differences on performance between the black screen and the blurred screen were qualitatively observed or reported, and the blurred screen seemed to be a static image). For each sentence, the signal-to-noise ratio was pseudo-randomly picked from 4 to 12 values, that were selected individually based on the results from the adaptive tracking procedure.

In the visual-only block, the video fragments of the female speaker were shown on the screen together with the acoustic background noise [at 60 dB(A)] and without auditory speech signal. For each sentence, the standard deviation of the Gaussian blurring kernel of the video images was pseudo-randomly picked from 5 to 10 values; the five most common values were 0, 6, 12, 16, and 20 pixels both for normal-hearing participants and CI users.

Notably, the trials in these two blocks contained a single unisensory informative signal (auditory vs. visual) in the presence of an uninformative stimulus from the other sensory modality. This makes these trials similar to the unisensory trials from the second experiment regarding sensory stimulation (see next section).

To avoid priming effects of sentence content (but not word content), a sentence was never repeated within a block. For each participant a different set of random signal-to-noise ratios, spatial blurs, and sentence permutations were selected. Importantly in this experiment, participants should focus on one sensory modality, and ignore the other, in order to reach maximum performance.

### Divided-Attention Task

In this experiment, audiovisual sentences (80–120 trials) were presented in one block. This experiment was conducted on another day than the focused-attention experiment. For each sentence, a visual blur and an auditory signal-to-noise ratio were chosen in pseudo-random order from 5 values, yielding 25 audiovisual stimulus combinations, selected in pseudo-random order. These values were selected individually based on the performance in the focused-attention experiment. We aimed for a unisensory speech-recognition performance of 0, 25, 50, and 75% for each participant, but as the maximum performance did not always reach 75%, other values were then chosen by the experimenter. The most common values were the same as for the previous experiment. In the unisensory trials of this task, the visual blur was extreme with a standard deviation of 70 pixels for the acoustic-only trials (this blur led to a subjective percept of static image), and the visual-only trials only contained auditory noise and no signal. Importantly, in contrast to the focused-attention task, participants could use information from both the auditory and visual modality in order to recognize words throughout most of the experiment, although some sentences were only informative in one sensory modality, but not in the other due to either extreme visual blurring (70-pixel blur) or absence of an acoustic signal.

### Data Analysis

**FIGURE 2:**
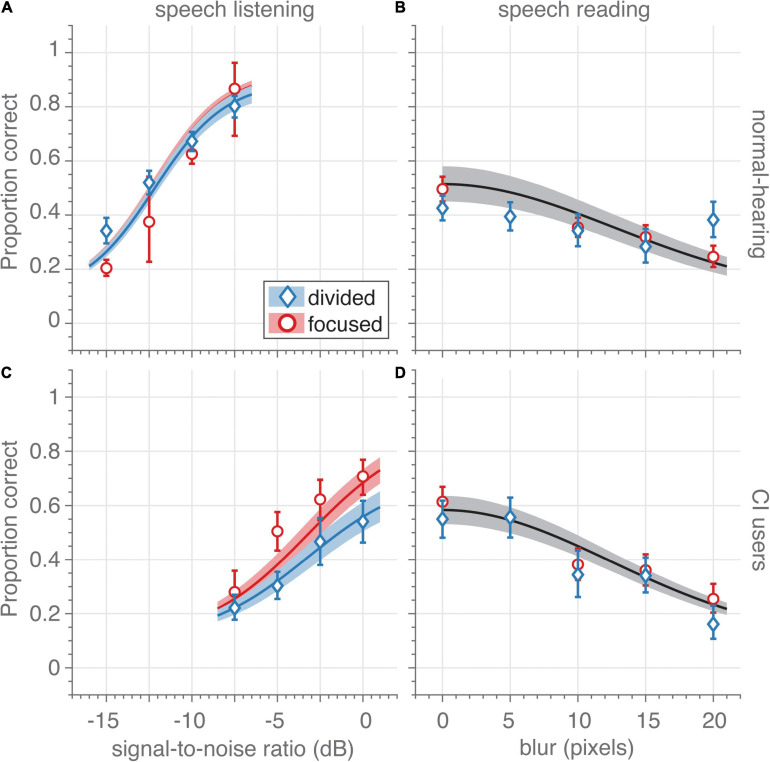
Unisensory speech recognition. **(A,C)** Auditory-only speech recognition (proportion correct) as a function of signal-to-noise ratio (dB) for **(A)** normal-hearing participants (*n* = 14) and **(C)** CI users (*n* = 7) in the focused- (red circles) and divided-attention (blue diamonds) tasks for auditory-only trials (visual blur is 70 pixels). The trials in the focused-attention sessions contained informative stimuli of a single modality, while in the divided-attention task, trials with auditory, visual and audiovisual informative stimuli were randomly interleaved. Only data from unisensory auditory and visual trials are shown in these figures. Note that although the unisensory stimuli were the same for both tasks, CI users recognized more auditory words correctly in the focused-attention task (red) than in the divided-attention task (blue). This effect was absent for the normal-hearing participants. **(B,D)** Visual-only speech recognition as a function of spatial blur (in units of pixel standard deviations) for **(B)** normal-hearing participants and **(D)** CI users in the focused- (red circles) and divided-attention (blue diamonds) tasks for visual-only trials (no auditory signal is presented). Note that due to the large similarity in visual recognition scores for both tasks, a single psychometric curve was fitted through the combined data (black curve and patch). Symbols and bars indicate mean and 95%-confidence intervals, respectively, of the raw data (proportion correct) pooled across participants. The data were binned to four signal-to-noise ratios and five blurs (as indicated by the abscissa value) for graphical purposes. Curves and patches indicate means and 95%-HDI, respectively, of the psychophysical-function group-level fits.

**FIGURE 3:**
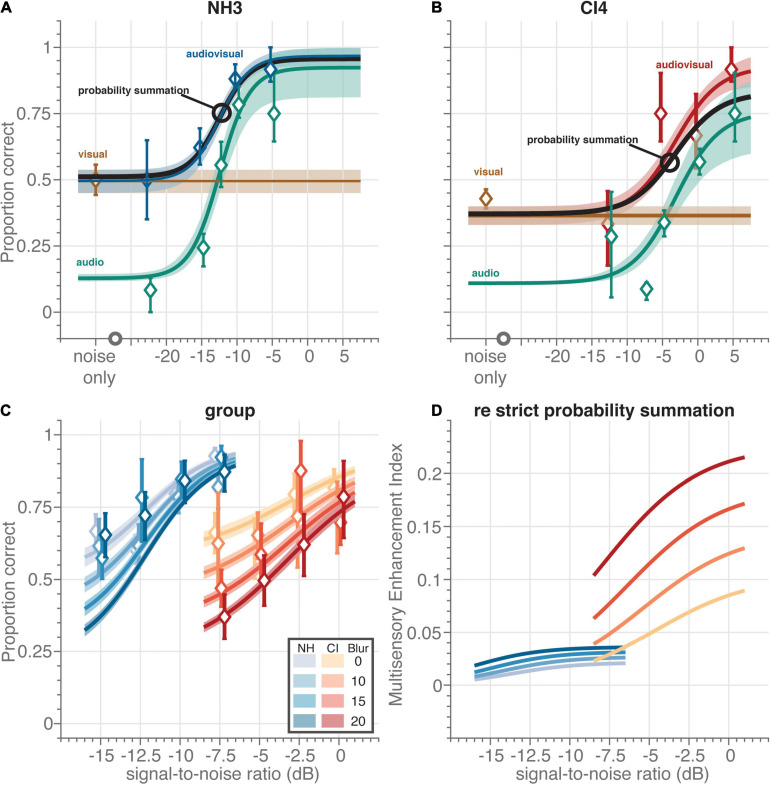
Multisensory speech recognition. Individual data and fit for **(A)** normal-hearing (NH) participant NH3 and **(B)** CI user CI4. All data was obtained from the divided-attention task. For the visual and audiovisual sentences, the video blur was 10 pixels. Symbols and bars indicate mean and 95%-confidence intervals, respectively, of the raw data (proportion correct) pooled across participants. The data was obtained (by definition) from the divided-attention task. Curves and patches indicate means and 95%-HDI, respectively, of the psychophysical-function population fits. For comparative purposes, we show the fitted speech reading performance level as a horizontal brown line. **(C)** Audiovisual speech recognition scores as a function of acoustic signal-to-noise ratio (dB) for normal-hearing participants (blueish diamonds) and CI users (reddish diamonds) for four blur values (as indicated by contrast). **(D)** Multisensory enhancement index (MEI) as a function of acoustic signal-to-noise ratio (dB) for normal-hearing participants (blue colors) and CI users (red colors) for four blur values (as indicated by contrast). The MEI quantifies the multisensory enhancement of the trade-off model over strict probability summation.

For graphical purposes, the proportion of words correct responses are plotted in raw form pooled across participants for each group as mean and 95%-HDI in Figures 2, 3 for the most common signal-to-noise ratios and blurs. For quantitative, statistical analysis, we evaluated psychometric functions fitted to data (as explained in the next sections), rather than directly testing the raw psychophysical results (correct answer- and lapse-rates) by means of conventional significance tests, such as ANOVA. The primary reason for this, is that the psychometric functions represent a suitable model for the data at hand; e.g., one can include a monotonic relationship with signal-to-noise ratio and blur, the model includes a binomial response distribution, it allows for the inclusion of existing, quantitative models of audiovisual integration, and one can fit all (uni- and multisensory) data with the same model. Conventional models have to be modified extensively and/or need to have their data transformed, both of which are non-trivial.

### Unisensory Psychometric Functions

To relate each participant’s responses to the intensity of the unisensory stimuli (i.e., auditory signal-to-noise ratio or visual blur), *x*, we fitted a psychometric function *F* to the unisensory data, the shape of which depended on the sensory modality, *m* (auditory vs. visual). For the auditory-only data, a logistic function was fitted (Kuss et al., 2005; van de Rijt et al., 2019):

(1) $$F_{A}\,\left(x_{A};\theta_{A},\omega \right)={\left(1+{\text{e}}^{\left(-\frac{2\,\text{ln}\,9}{\omega }\,\left(x_{A}-\theta_{A}\right)\right)}\right)}^{-1}$$

where *F*_*A*_(*x*_*A*_;θ_*A*_;ω_*A*_) characterizes the change in auditory word recognition rate as a function of the auditory signal-to-noise ratio, *x_A*; θ_*A*_ is the auditory recognition threshold, the signal-to-noise ratio at which the words are expected to be correctly recognized in 50% of the time; and ω_*A*_ is the width of auditory recognition function, the signal-to-noise ratio range in which *F_A* ranges from 0.1 to 0.9.

For the visual-only data, an exponential function *F_V* was taken with only a single parameter:

(2) $$F_{V}\,\left(x_{V};\theta_{V}\right)={\text{e}}^{-\frac{x_{V}^{2}}{\theta_{V}^{2}}}$$

where *F*_*V*_(*x*_*V*_;θ_*V*_) characterizes the change in visual word recognition rate as a function of the visual blur, *x_V*; θ_*V*_ is the visual recognition threshold, the blur at which the words are expected to be correctly recognized in 36.8% of the time, i.e., for *x*_*V*_=θ_*V*_. The blurs *x*_*V*_ and visual threshold θ_*V*_ are only defined for values larger than or equal to 0.

The exact shape of the functions of Eqs. 1, 2 are chosen slightly arbitrarily. However, both functions (Eqs. 1, 2) have a sigmoidal shape that is typical for psychometric data, both have few free parameters (2 and 1), fitted their corresponding data well (i.e., Figure 1), and yielded meaningful parameter values (i.e., a positive visual threshold).

### Lapse

To infer the probability of correct-word recognition Ψ, we included a lapse probability, λ, to the psychometric function *F* for both modalities *m*:

(3) $$\Psi_{m,e}=\left(1-\lambda_{m,e}\right)\,F_{m}$$

The lapse probability, λ, accounted for the less-than-perfect recognition probability for visual words without blurring and for auditory words at the highest signal-to-noise ratios, both for the CI users and the normal-hearing participants. With probability *λ*_(*m*,*e*)_ a participant has a momentary lapse (i.e., makes a choice independent of stimulus intensity) for modality *m* during experiment *e* (focused-attention vs. divided attention). With probability (1−*λ*) the participant does not have a lapse and has a chance of *F_m* to give the correct answer. The lapse probability could reflect several issues: e.g., a momentary lapse of attention, blinking during the visual trials, or the lack of increase in information with increasing stimulus intensity due to for example processing issues of the cochlear implant.

Crucially, the estimate for the lapse probability was, at first, inferred separately for the experimental tasks (focused-attention vs. divided), as we hypothesized that the separate tasks could differentially affect attentional demands, potentially leading to observed differences in attentional lapses.

We modified this slightly, as we observed no significant differences in the visual lapse probability between experimental tasks (Figure 2). Thus, the final fitted model (Eqs. 1–3), as reported here, included the auditory lapse probability as the only parameter that was free to vary between experimental tasks. Constraining the model in such a way had no effect on the conclusions.

### Multisensory Psychometric Function Defined by Probability Summation

We modeled the audiovisual speech recognition as a mere statistical-summation effect that is distinct from true neural audiovisual integration. In this model of probability summation (see section “Introduction”), participants recognize a word from either the auditory-only or the visual-only condition, which are considered independent processing channels. Thus, if a subject fails to recognize a word from either one of the modalities, the probability of failure is (1−Ψ_*A*_)×(1−Ψ_*V*_). It then follows that the probability of word recognition in the presence of the two modalities without integration is given by:

$$\Psi_{s\,u\,m}=1-P_{f\,a\,i\,l}$$

(4) $$=1-\left(1-\Psi_{A}\right)\times \left(1-\Psi_{V}\right)=\Psi_{A}+\Psi_{V}-\Psi_{A}\times \Psi_{V}$$

where Ψ_*sum*_ is the probability to successfully recognize a word according to the summation model, Ψ_*A*_ is the probability to recognize an auditory word in the auditory-only condition, and Ψ_*V*_ is the probability of recognizing a visual word. From this, one can observe that having both modalities available, rather than one, automatically increases the probability of stimulus recognition.

We chose to fit this model because previous evidence (van de Rijt et al., 2019) showed that speech recognition of the audiovisual materials could be described well by probability summation. Importantly, the data was accurately fitted by this model (see section “Model Selection”), with one caveat: the fit was better if the lapse probabilities for the audiovisual stimuli (by definition, only presented in the divided-attention task) were estimated from the unimodal lapse probabilities as found in the focused-attention task, rather than from the divided-attention task.

This meant that this model could only predict an enhancement of speech recognition for multisensory stimuli through a combination of mere statistical facilitation and a change in auditory lapse probability across experimental tasks. To visualize this (Figure 3D), we determined the multisensory enhancement index (MEI):

(5) $$\text{MEI}=\frac{\Psi_{t\,r\,a\,d\,e-o\,f\,f}}{\Psi_{s\,t\,r\,i\,c\,t}}-1$$

with Ψ_*strict*_ and Ψ_*trade*−*off*_ being the probability to successfully recognize a word according to the summation model with an auditory lapse probability taken from the divided-attention (strict) and focused-attention (trade-off) tasks, respectively. An MEI close to zero is in line with statistical facilitation, and no change in lapse probability. Positive values are evidence for an observed multisensory enhancement and an increased auditory lapse probability.

### Guess Probability

We also included a guess rate of 10% that accounts for a fixed probability of 0.1 of correctly choosing 1 of the 10 alternatives by chance alone (0.9Ψ+0.1). This was the same for every participant, modality and experimental task, as it depended on the design of the Matrix test itself.

### Approximate Bayesian Inference

Parameter estimation was performed using approximate Bayesian inference. The models described by Eqs. 1–4 were fitted on all data simultaneously. The parameters were estimated for every participant, which depended on the estimation of overarching group parameters, separately for the normal-hearing participants and CI users, in a hierarchical fashion.

The estimation procedure relied on Markov Chain Monte Carlo (MCMC) techniques. The estimation algorithms were implemented in JAGS (Plummer, 2003) through matJAGS (Steyvers, 2011). Three MCMC chains of 10,000 samples were generated. The first 10,000 samples were discarded as burn-in. Convergence of the chains was determined visually, by checking that the shrink factor $\hat{R}$ is less than 1.1 and by checking that the effective sample size is larger than 1,000 (Gelman et al., 2013). From these samples of the posterior distributions, we determined the mean and the 95%-HDI as a centroid and uncertainty estimate of the parameters, respectively.

### Model Selection

To test for the appropriateness of the models in Eqs. 1–4, we compared them against less-restrictive models. To that end, we performed a qualitative check via visual inspection (c.f. Figures 1, 2), but we also quantitatively determined the Bayesian Information Criterion (BIC) for each model:

(6) $$\text{BIC}=\text{ln}\,\left(n\right)\,k-2\,\text{ln}\,\left(\hat{L}\right)$$

where *k* denotes the number of parameters of the model, *n* the number of samples and $\hat{L}$ the maximized value of the binomial likelihood function.

## Results

### Overview

Fourteen normal-hearing participants and seven post-lingually deaf unilaterally implanted CI users were asked to identify words (Matrix test) (van de Rijt et al., 2019) presented acoustically and/or visually. We varied task difficulty in both experiments, by blurring the video, and by presenting acoustic background noise at several levels. The sentences were either presented in two separate unisensory blocks, or in one large randomized uni- and multisensory block, which we termed the focused- and divided-attention experiments, respectively. In the focused-attention experiment (Figure 2; red), the sentences were either presented in an acoustic-only block (Figures 2A,C, red circles), or in a visual-only block (Figures 2B,D, red circles), and the participant could *focus* solely on listening or lipreading, respectively. In the divided-attention experiment auditory (Figures 2A,C, blue diamonds), visual (Figures 2B,D, blue diamonds) and audiovisual (Figure 3) sentences were presented in pseudo-random order, all interleaved in one block. Importantly, in each trial of the divided-attention task, participants had no prior knowledge on how informative each modality would be. In this task, participants were free to focus on one modality, or to *divide* attention across both modalities.

To estimate parameters of interest, such as the signal-to-noise ratio and blur at which performance level was 50% and (attentional) lapse probabilities, we fitted psychophysical-function curves through the data (as fully explained in the section “Materials and Methods”). We report on the mean and 95%-highest-density interval (HDI) of the fitted estimate distributions of the group-level parameters, and show both the fitted curves for each group and the data averaged across participants in the figures.

### Unisensory Speech Perception

When sentences were presented only acoustically (with no visual information) (Figures 2A,C), the two groups clearly differed in their ability to recognize words, as expected. Typically, the normal-hearing participants (Figure 2A) recognized 50% of the words correctly in the unisensory hearing condition at a signal-to-noise ratio (auditory threshold, Eq. 1) of –12 dB (HDI = [–12.4,–11.5] dB) vs. –3.1 dB for the CI users (HDI = [–4.4, –1.7] dB, Figure 2C), independently of whether the unisensory trials were embedded amongst multisensory trials or not (i.e., for both the divided-attention and focused-attention tasks, blue and red). For both participant groups, the proportion of correctly recognized words strongly depended on the actual signal-to-noise ratio; to increase performance levels from 5– to 95%-word recognition (psychometric curve width), the signal-to-noise ratio needed to be increased by 7.4 dB on average for the normal-hearing participants (HDI = [6.5, 8.5] dB; Figure 2A) and slightly more for CI users by on average 10.4 dB (HDI = [8.8, 12.2] dB; Figure 2C). As expected, both these results confirm that listening in background noise for CI users is considerably more difficult than for normal-hearing participants.

The parameter of main interest in this study is the lapse probability (Eq. 3), i.e., the probability of not recognizing words even at the highest signal-to-noise ratio and without blur. Lapses occurred even in the focused-attention task as evidenced by the non-perfect saturation performance at the highest signal-to-noise ratios; the average performance of normal-hearing participants and CI users saturated at around 90 and 84% correct, respectively (Figures 2A,C, red; HDI = [85, 94] and [74, 92%]). A larger lapse probability for the CI users compared to the lapse probability for the normal-hearing participants may be expected due to technical limits of the cochlear implant and the maximal comfortable loudness levels experienced by the CI users, but note that evidence for any difference was actually small (mean 5%, HDI = [–5, 17]%).

More importantly and more clearly, in the divided-attention task the CI users recognized 22% (HDI = [6.7, 38]%) fewer words than in the focused attention task (Figure 2C, blue vs. red). This difference was not clearly evident for the normal-hearing participants (mean difference 3.9%, HDI = [–4.0, 14]%; Figure 2A, blue vs. red). Evidence for group differences in auditory lapse probability during the divided-attention experiment was substantial (on average, the lapse probability for normal-hearing participants was 24% lower than for the CI users, HDI = [8, 41]%).

When sentences were presented only visually (with no acoustic information) (Figures 2B,D), the proportion of correctly recognized words depended on the amount of blur, and were largely similar for both groups; the visual threshold (i.e., the blur at 36% of the maximal lipreading performance, Eq. 2 was on average 17.7 and 18.3 pixels for CI users (Figure 2D) and normal-hearing (Figure 2B) participants, respectively (HDI = [16.0, 19.7] and [15.2, 21.8] pixels, respectively) for both tasks. Of course, lipreading abilities were far from perfect even without blurring.

No major difference in lipreading performance was observed for the visual lapse probability, so we pooled the data from both tasks to estimate this parameter. Normal-hearing participants (Figure 2B) had a lapse in word recognition in 54% of the cases (HDI = [42, 65]%), while CI users (Figure 2D) incorrectly recognized unblurred visual words in 46% of the cases (HDI = [36, 56]%). While one may expect CI users to be better lip-readers than normal-hearing participants, differences between groups were actually small (on average 8%, HDI = [–8, 23]%).

In summary, largely in contrast to the normal-hearing participants, the CI users experienced more speech-recognition problems when attention had to be divided between more than one sensory modality. These problems were especially conspicuous for listening, the sensory modality that faced the largest difficulties for the CI users.

### Multisensory Integration

We next analyzed whether speech perception of audiovisual stimuli would be enhanced for both groups of participants in the divided-attention task (Figure 3). Figures 2A,B show examples of individual participants (NH3 and CI4) in the divided-attention task at a visual blur of 10 pixels. The unisensory data and fits for these two participants (Figures 2A,B brown and blue for speech reading and listening, respectively) are in line with the group-level data and fits as described in the previous section (cf. Figure 2, blue). The audiovisual speech recognition (Figure 3A, blue and Figure 3B red for NH3 and CI4, respectively) outperforms or equals either unimodal speech recognition; for very low and high signal-to-noise ratios, audiovisual performance tends to equal visual or auditory performance. For intermediate signal-to-noise ratios, audiovisual performance is clearly enhanced. Such an enhancement of multisensory performance could potentially be due to mere statistical facilitation, if the participants would recognize a word by using either the available auditory, or visual information, without actually integrating both inputs. The percept is then determined by whichever sensory channel wins the race (probability summation) (van de Rijt et al., 2016, 2019; Colonius and Diederich, 2017). The audiovisual enhancement would then be fully determined by the unisensory auditory and visual recognition performance during the divided-attention task. To check for this possibility, we compared the data to the prediction from this probability-summation model (Figures 3A,B, black curve, see section “Materials and Methods”). For the normal-hearing participant (Figure 3A; cf. black markers and blue curve), the model’s prediction corresponded quite well to the data. Hence, despite the improvement in audiovisual recognition rates, the normal-hearing participant did not seem to benefit from multisensory integration. In contrast, although the CI user evidently had difficulty to recognize a pure auditory speech signal in the multisensory divided-attention task (Figure 3B, blue; note the increased threshold and the larger lapse probability compared to the data shown in Figure 3A), they outperformed the probability-summation model for the combined audiovisual speech signals by about 10% at the highest signal-to-noise ratios (Figure 3B, compare red vs. black curves).

We quantified the audiovisual performance for all participants of both groups (visualized as a function of the acoustic signal-to-noise ratio for four different magnitudes of visual blur, Figure 3C) by fitting a probability-summation model that was fully determined by the unisensory auditory and visual recognition performance (Eqs. 1–4). Typically, the observed multisensory enhancement should be compared to probability-summation of unisensory performance obtained from the same experimental regime, which in the current experiment would be from the divided-attention task. We term this model the strict probability-summation model, and it contains only parameters (such as the auditory lapse probability) that are estimated from the divided-attention task to predict audiovisual speech recognition scores. In Figure 3C, we show the results of an alternative model, that actually captures the multisensory enhancement by using the unisensory data obtained during the focused-attention task. We did this because the increased lapse probability for listening by the CI users in the divided-attention task over the focused-attention task (Figure 2C) appeared to reflect the multisensory enhancement over the strict probability-summation model (e.g., Figure 3B, compare the red fit curve to the black curve). Participants basically seem to trade-off their ability to focus for a multisensory enhancement, which is why we designate this model the trade-off model. In essence, the difference in recognition scores between the two tasks was captured by the difference in auditory lapse probability, the single alternative model parameter free to vary between tasks.

Nevertheless, the trade-off model describes the data for both tasks quite well (Table 1, see section “Materials and Methods,” and Figures 2A,B). Note that the pooled data generally appear to be at higher performance levels than the group-level fits of the trade-off model, at least for the normal-hearing participants (Figure 3C, blue). This follows from the fact that we individualized the stimulus parameters for each participant; the data was obtained at lower signal-to-noise ratios and higher blurs more often for the better performers. The group-level fits better describe the expected overall group performance through extrapolation to a larger range of signal-to-noise ratios and blurs. By comparing the fits to the audiovisual data (Figure 3C) to the unisensory fits (cf. Figure 2), one can observe that audiovisual speech recognition is better than unisensory speech recognition; even at a blur of 20 pixels and a signal-to-noise ratio of –15 dB for the normal-hearing and of –7.5 dB for the CI users (around 0.2 vs. 0.35 for unisensory and multisensory stimulation, respectively).

**TABLE 1:** Model comparison.

		**Normal-hearing**	**CI users**
ΔBIC	Trade-off	0	0
	Strict	12	35
R^2^	Trade-off	0.89	0.78
	Strict	0.89	0.75
Mean signed error	Trade-off	0.00	0.01
	Strict	0.00	0.05

*The relative Bayesian Information Criterion ΔBIC, the coefficient of determination R^2^, and mean signed error are shown for both the trade-off and strict probability-summation model for both participant groups. All measures indicate the trade-off model performs better for the CI users than the strict model. For the normal-hearing participants, the ΔBIC favors the trade-off model, while the other two measures indicate there is no difference.*

To illustrate the benefits of multisensory stimulation more clearly, we determined the multisensory enhancement index (MEI, Eq. 5). This index quantifies by how much multisensory performance of the trade-off model was improved over the strict probability-summation model (Figure 3D). A MEI close to zero is in line with strict statistical facilitation, while positive values are evidence for audiovisual enhancement due to multisensory integration. The index shows marginal improvement for the normal-hearing group (between 0.005 and 0.036, depending on signal-to-noise ratio and blur, Figure 3D), and a far more prominent benefit for CI users that was about 4–6 times larger (0.023–0.22). A larger MEI for lower-informative stimuli or poorer-performing individuals would be evidence for inverse effectiveness (Stein and Meredith, 1993; Bremen et al., 2017). This effect seemed to occur for the groups and the blurs; CI users exhibited more enhancement than the normal-hearing participants (Figure 3D, red vs. blue) and the relative multisensory improvements were largest for the highest blurs (Figure 3D, e.g., the MEI for the 0-pixel blur was lower than for the 20-pixel blur, especially for the CI users). In contrast, for acoustic information a direct, rather than an inverse, relationship was observed: the lowest signal-to-noise ratios elicited the smallest enhancements (Figure 3D, the MEI curves all decline for lower signal-to-noise ratios).

## Discussion

### Summary

Results show that CI users benefit from multisensory integration in a divided-attention task (Figure 3), but that their unisensory performance under such conditions deteriorates when compared to listening under focused attention (Figure 2). Interestingly, their multisensory benefit matches the prediction obtained from probability summation of their (better) focused-attention performance (Figure 3). In contrast, the normal-hearing participants do not have poorer unisensory performance in a divided-attention task, and their multisensory scores are accounted for by strict probability summation. Normal-hearing participants reached higher auditory recognition scores than the CI users. As expected, these results confirm the well-known fact that listening for CI users is considerably more difficult. Factors that likely contribute to the difficulties in understanding auditory speech in noise environments are the lack of access to finely detailed spectral and temporal information and a limited dynamic range (Friesen et al., 2001). In contrast, CI users and normal-hearing participants had similar lipreading skills (Figures 2B,D). This was slightly unexpected, as others have reported better lipreading abilities by CI users (Bernstein et al., 2000; Rouger et al., 2007). The current experiment, however, entailed recognition of a limited closed-set matrix sentences of five words with 10 options per word. This potentially makes lipreading for normal-hearing individuals, who might be unaccustomed to lipreading in general, easier than in open sets with many more alternatives. Also, both the CI users and normal-hearing participants do have normal vision. As such, one might perhaps expect similar visual, lipreading skills.

### Individual vs. Group Performance

We have focused on a group-level analysis, even though it is well-known that performance levels can vary widely across individuals, both for normal-hearing lip-reading abilities (van de Rijt et al., 2019) and for speech-listening abilities of CI users. We do present individualized signal-to-noise ratios and blurs, and our fit procedures do account for individual variability. However, we feel that due to the limited number of participants, our data does not allow to draw conclusions on whether idiosyncratic unisensory perceptual abilities influence multisensory integration or attention. This, of course, would be highly relevant for individualized counseling of CI users.

Note that the pooled data generally conform well to the group level fits, but may be off for certain conditions (i.e., Figure 2C, red–CI speech listening; Figure 3C, blue–normal-hearing, multisensory speech recognition). As mentioned in the Results, this follows from individualized stimulus parameters for each participant; the pooled data was influenced by the individual data of the better performers at the lower signal-to-noise ratios and higher blurs, and therefore may indicate better performance than the group-level fit lines. The individual fit always conformed well to its corresponding data (e.g., Figures 3A,B and Table 1).

### Age Mismatch

The largest confound in this study is the age difference between the groups. We chose to have a participant group with normal, optimal hearing and sight to contrast to the CI group. This led to the inclusion of younger individuals in the normal-hearing group compared to the CI group, since elderly individuals have a high probability of suffering from presbycusis. As such, observed differences between groups may be due to either sensory deficits that are different for each group or attentional deficits that vary with age. Irrespective of which deficit underlies the observed differences between groups, we feel that the results for the CI users are still highly relevant, as they typically are older when receiving a CI.

### Attentional Lapse in Unisensory Performance

Cochlear implant users missed fewer words when they could focus on listening alone (in the focused-attention task, Figure 2C) than in situations with uncertainty about the modality of the upcoming stimulus (in the divided-attention task). Note that this is precisely the sensory condition of every-day life. This may suggest that due to impoverished sensory information more effort is required by CI users to be able recognize speech at higher performance levels. However, the extra effort cannot be maintained by CI users if attention has to be spread out across multiple, potentially informative sensory modalities. The CI users seem to have reached the limits of attentional resources in the divided-attention task. These limits are not reached when sensory information is not impoverished, i.e., for normal-hearing individuals and for lipreading 1A, B, D; lapse probabilities are similar across tasks). To be clear, this does not necessarily imply that CI users have less attentional resources than normal-hearing individuals; they need to address those resources more. Of course, due to the rather advanced ages of the CI users, such a difference between groups might not be surprising. However, in an earlier study by Tye-Murray et al. (2016), the multisensory benefit in speech recognition seemed entirely driven by age-related changes in auditory and visual abilities. Note that this is in line with our observations that only the auditory lapse probability significantly changed across tasks for the CI users, and that visual performance remained the same. This may suggest that the group differences are not due to general-attention mechanisms, but are related specifically to the impaired sense.

### Multisensory Integration

Following this line of reasoning, one may wonder why CI users attempt to lipread at all. Barring any other benefits, the optimal decision would be to focus on the most-informative sensory modality, and ignoring the other. Even for CI users, listening is generally (i.e., in quiet environments) the far better modality for the purposes of speech recognition. Probabilistic, uninformed switching between listening and lipreading would lead to an overall worse performance (Ege et al., 2018). One benefit to offset this drawback could be that switching enables individuals to scan the specific environment and determine whether listening or lipreading would be the most informative modality for the given situation (Berniker et al., 2010; Ege et al., 2019). Obviously from the current experiments, another benefit could be that the detriment in listening is accompanied by an enhancement of speech recognition for multisensory stimuli. Indeed, although CI users had poorer unisensory recognition scores in the divided-attention task than in the focused attention task (Figure 2), they outperformed the strict probability-summation model (Figure 3D). Conversely, the normal-hearing individuals do follow strict probability summation (van de Rijt et al., 2019). Because of this, CI users appear to be better multisensory integrators than the normal-hearing individuals (Rouger et al., 2007; Figure 3D).

### Integration-Attention Trade-Off

Intriguingly, the trade-off model suggests that the exact compensation of the listening decline (Figure 2C) by multisensory enhancement (Figure 3D) may be explained by an integration-attention trade-off mechanism for CI users. To benefit from multisensory integration, attention needs to be divided across all relevant signals. Only then will integration be able to enhance source identification and selection by filtering out irrelevant noise sources. The cost of this benefit is the decline in attentional amplification of unisensory signals. In our model, this is fully and solely captured by the change in auditory lapse probability (Eq. 3), which amounted to be about 22% on average for CI users (see section “Results”–subsection “Unisensory Speech Perception”). The multisensory enhancement seems inversely proportional to this increase in lapses. This inverse proportionality was captured by the trade-off probability-summation model (Eqs. 4, 5) that used the lapse rates in the focused-attention task rather than in the divided-attention task. A direct comparison between multisensory enhancement and the change in auditory lapse probability is impossible, as enhancement depends on the strength of the visual and acoustic signals (Figure 3D), while the lapse rate does not. However, in the ideal case, i.e., for the weakest visual signals and strongest auditory signals, the multisensory enhancement should equal the lapse rate difference in magnitude for the weakest visual signals and strongest auditory signals; note that the MEI is 0.22 for the highest blur at a signal-to-noise ratio of 0 dB.

## Conclusion

Normal-hearing participants can attend extensively on auditory and visual cues, while (post-lingually deaf) CI users need to divide their attentional resources across modalities to improve multisensory speech recognition–even though this leads to a degradation in unisensory speech recognition. We argue that in order to determine the acoustic benefit of a CI toward speech recognition *per se*, situational factors need to be discounted by presenting speech in realistic audiovisual environments. If in every-day life, no reliable prior information is available on the upcoming presence of auditory, visual or audiovisual speech, then CI users would be better off to avoid lipreading and make do with what they hear.

## Author’s Note

Deaf individuals using a cochlear implant require significant amounts of effort to listen in noisy environments due to their impoverished hearing. Lipreading can benefit them and reduce the burden of listening by providing an additional source of information. Here we show that the improved speech recognition for audiovisual stimulation comes at a cost, however, as the cochlear-implant users now need to listen and speech-read simultaneously, paying attention to both modalities. The data suggests that cochlear-implant users run into the limits of their attentional resources, and we argue that they, unlike normal-hearing individuals, always need to consider whether a multisensory benefit outweighs the unisensory cost in everyday environments.

## Data Availability Statement

The original contributions presented in the study are publicly available. These data can be found here: https://doi.org/10.34973/jy8p-dw52.

## Ethics Statement

The studies involving human participants were reviewed and approved by the Ethics Committee of Arnhem-Nijmegen. The patients/participants provided their written informed consent to participate in this study. Written informed consent was obtained from the individual(s) for the publication of any potentially identifiable images or data included in this article.

## Author Contributions

LR, AO, and MW designed the research and wrote the manuscript. LR performed the research. MW analyzed the data. LR and MW drafted the initial research concept. All authors contributed to the article and approved the submitted version.

## Conflict of Interest

The authors declare that the research was conducted in the absence of any commercial or financial relationships that could be construed as a potential conflict of interest.

## Publisher’s Note

All claims expressed in this article are solely those of the authors and do not necessarily represent those of their affiliated organizations, or those of the publisher, the editors and the reviewers. Any product that may be evaluated in this article, or claim that may be made by its manufacturer, is not guaranteed or endorsed by the publisher.
